# Diethyl 2-[(1-methyl-1*H*-pyrrol-2-yl)methyl­ene­amino]-5-(2-thienylmethyl­ene­amino)thio­phene-3,4-dicarboxyl­ate

**DOI:** 10.1107/S160053680800799X

**Published:** 2008-04-02

**Authors:** Stéphane Dufresne, W. G. Skene

**Affiliations:** a Department of Chemistry, University of Montreal, CP 6128, succ. Centre-ville, Montréal, Québec, Canada H3C 3J7

## Abstract

Both imine bonds of the title compound, C_21_H_21_N_3_O_4_S_2_, were found to be in the *E* configuration. The terminal pyrrole and thio­phene rings are twisted by 2.5 (3) and 2.3 (2)°, respectively, from the mean plane of the central thio­phene to which they are attached. The structure is disordered by exchange of the terminal heterocyclic rings; the site occupancy factors are *ca* 0.8 and 0.2. The crystal packing involves some π–π stacking [3.449 (4) Å between pyrrole and terminal thio­phene rings].

## Related literature

For general background, see: Dufresne *et al.* (2007 ▶). For a similar compound, see: Dufresne *et al.* (2006 ▶).
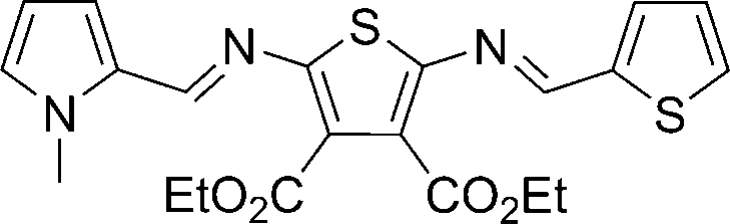


## Experimental

### 

#### Crystal data

C_21_H_21_N_3_O_4_S_2_
*M* *_r_* = 443.53
Monoclinic, 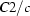

*a* = 30.7355 (14) Å
*b* = 6.9617 (4) Å
*c* = 19.5163 (9) Å
β = 92.732 (2)°
*V* = 4171.2 (4) Å^3^
*Z* = 8
Cu *K*α radiation
μ = 2.60 mm^−1^
*T* = 150 (2) K
0.14 × 0.09 × 0.05 mm

#### Data collection

Bruker SMART 6K diffractometer
Absorption correction: multi-scan (*SADABS*; Sheldrick, 1996 ▶) *T*_min_ = 0.712, *T*_max_ = 0.881
24275 measured reflections
4076 independent reflections
3330 reflections with *I* > 2σ(*I*)
*R*_int_ = 0.041

#### Refinement

*R*[*F* ^2^ > 2σ(*F* ^2^)] = 0.041
*wR*(*F* ^2^) = 0.115
*S* = 1.03
4076 reflections
394 parameters
544 restraints
H-atom parameters constrained
Δρ_max_ = 0.28 e Å^−3^
Δρ_min_ = −0.38 e Å^−3^

### 

Data collection: *SMART* (Bruker, 2003 ▶); cell refinement: *SMART*; data reduction: *SAINT* (Bruker, 2004 ▶); program(s) used to solve structure: *SHELXS97* (Sheldrick, 2008 ▶); program(s) used to refine structure: *SHELXL97* (Sheldrick, 2008 ▶); molecular graphics: *ORTEP-3* (Farrugia, 1997 ▶); software used to prepare material for publication: *UdMX* (Marris, 2004 ▶).

## Supplementary Material

Crystal structure: contains datablocks I, New_Global_Publ_Block. DOI: 10.1107/S160053680800799X/fl2192sup1.cif

Structure factors: contains datablocks I. DOI: 10.1107/S160053680800799X/fl2192Isup2.hkl

Additional supplementary materials:  crystallographic information; 3D view; checkCIF report

## Figures and Tables

**Fig. 1.**
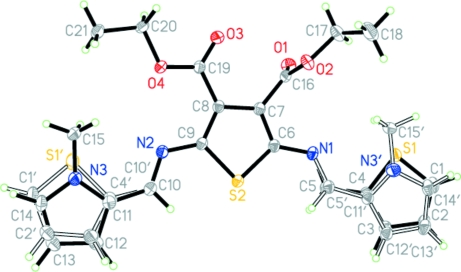
ORTEP representation of (I) showing the disorder present in the terminal rings with the numbering scheme adopted (Farrugia, 1997). Ellipsoids drawn at 30% probability level.

**Fig. 2.**
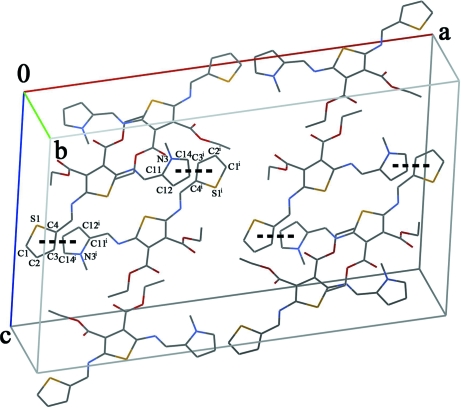
Supramolecular structure showing the intermolecular π-stacking giving the structural arrangement. Dashed lines indicate the π-stacking. The H atoms and disorder was omitted for clarity. [Symmetry codes: (none) x, 1 - y, -1/2 + z; (i) 1/2 - x, -1/2 + y, 1.5 - z.]

## supplementary crystallographic information

### Comment

The title comound (I), seen in Fig. 1, was synthesized during the course of our ongoing research relating to conjugated imines (Dufresne *et al.*, 2007). Despite the terminal heterounits of (I) being disordered by 18%, both imines were found to adopt the E isomer. Neither solvent nor counter-ions were found in the closed-packed stacking (Fig. 2).

A major point of interest is the imine bonds, 1.446 (9), 1.278 (2) and 1.377 (2)Å for C4—C5, N1—C5 and N1—C6, respectively. Similarly, the bond lengths for C10—C11, N2—C10 and N2—C9 are 1.423 (9), 1.299 (17) and 1.373 (2) Å. These distances are comparable to those fournd for an analogous all-thiophene compound, 1.441 (4), 1.272 (3) and 1.388 (3)Å (Dufresne *et al.*, 2006).

The terminal heteroaryl groups are slightly twisted from the mean plane of the central thiophene to which they are connected with a dihedral angle of 2.3 (2)° for the terminal thiophene and 2.5 (3)° for the *N*-methylpyrrole. The small dihedral angles show that the aryl groups of compound (I) are nearly coplanar. This is in contrast to its all-thiophene analogue whose comparable mean plane angles are 9.04 (4)° and 25.07 (6)°.

The crystal packing of (I) is also different from that found for the all-thiophene analogue. The molecules in the all-thiophene network are linearly aligned in one direction. In contrast, the molecules of (I) are misaligned by up to 16.84 (4)°. No traditional H-bonding was found, but π-π-stacking does occur as shown in Figure 2. π-π-stacking interactions occur between the pyrrole and the terminal thiophene of two different molecules, which are separated by 3.449 (4) Å.

### Experimental

In a 50 ml round bottom flask was added 1-methyl-2-pyrrole-carboxaldehyde (40 mg, 0.37 mmol) dissolved in 25 ml of anhydrous toluene to which was subsequently added 1,4-diazabicyclo[2.2.2]octane (159 mg, 1.42 mmol) and TiCl_4_ (0.28 ml, 0.28 mmol) as a 1.0 *M* solution in toluene at 0 °C followed by diethyl 2-((thiophen-2-yl)methyleneamino)-5-aminothiophene-3,4-dicarboxylate (100 mg, 0.28 mmol). The mixture was then refluxed for four hours followed by solvent removal. Purification by flash chromatography yielded the title product as a red solid (63 mg). The selected crystal was obtained by slow evaporation of a concentrated solution in acetone.

### Refinement

During the refinement, it became apparent that the structure was disordered as an inversion of the terminal heterocycles. We first tried to fix each part to half of the weight and then let it vary to the optimized proportion of 82:18. The temperature factors were less than desired because of the disorder requiring many constraints including fixing similar temperature factors and distances for every disordered atom. H atoms were placed in calculated positions (C—H = 0.95–0.98 Å) and included in the refinement in the riding-model approximation, with *U*ĩso~(H) = 1.2 *U*~eq~(C).

### Crystal data

**Table:**

C_21_H_21_N_3_O_4_S_2_	*F*_000_ = 1856
*M**_r_* = 443.53	*D*_x_ = 1.413 Mg m^−^^3^
Monoclinic, *C*2/*c*	Cu *K*α radiation λ = 1.54178 Å
Hall symbol: -C 2yc	Cell parameters from 25 reflections
*a* = 30.7355 (14) Å	θ = 15.0–30.0º
*b* = 6.9617 (4) Å	µ = 2.60 mm^−^^1^
*c* = 19.5163 (9) Å	*T* = 150 (2) K
β = 92.732 (2)º	Block, red
*V* = 4171.2 (4) Å^3^	0.14 × 0.09 × 0.05 mm
*Z* = 8	

### Data collection

**Table:**

Bruker SMART 6K diffractometer	4076 independent reflections
Radiation source: Rotating Anode	3330 reflections with *I* > 2σ(*I*)
Monochromator: Montel 200 optics	*R*_int_ = 0.041
Detector resolution: 5.5 pixels mm^-1^	θ_max_ = 71.9º
*T* = 150(2) K	θ_min_ = 2.9º
ω scans	*h* = −37→36
Absorption correction: multi-scan(SADABS; Sheldrick, 1996)	*k* = −7→8
*T*_min_ = 0.712, *T*_max_ = 0.881	*l* = −23→24
24275 measured reflections	

### Refinement

**Table:**

Refinement on *F*^2^	Secondary atom site location: difference Fourier map
Least-squares matrix: full	Hydrogen site location: inferred from neighbouring sites
*R*[*F*^2^ > 2σ(*F*^2^)] = 0.041	H-atom parameters constrained
*wR*(*F*^2^) = 0.115	*w* = 1/[σ^2^(*F*_o_^2^) + (0.0781*P*)^2^] where *P* = (*F*_o_^2^ + 2*F*_c_^2^)/3
*S* = 1.03	(Δ/σ)_max_ = 0.001
4076 reflections	Δρ_max_ = 0.28 e Å^−^^3^
394 parameters	Δρ_min_ = −0.38 e Å^−^^3^
544 restraints	Extinction correction: none
Primary atom site location: structure-invariant direct methods	

### Special details

**Table:**

Geometry. All e.s.d.'s (except the e.s.d. in the dihedral angle between two l.s. planes) are estimated using the full covariance matrix. The cell e.s.d.'s are taken into account individually in the estimation of e.s.d.'s in distances, angles and torsion angles; correlations between e.s.d.'s in cell parameters are only used when they are defined by crystal symmetry. An approximate (isotropic) treatment of cell e.s.d.'s is used for estimating e.s.d.'s involving l.s. planes.
Refinement. Refinement of *F*^2^ against ALL reflections. The weighted *R*-factor *wR* and goodness of fit *S* are based on *F*^2^, conventional *R*-factors *R* are based on *F*, with *F* set to zero for negative *F*^2^. The threshold expression of *F*^2^ > σ(*F*^2^) is used only for calculating *R*-factors(gt) *etc*. and is not relevant to the choice of reflections for refinement. *R*-factors based on *F*^2^ are statistically about twice as large as those based on *F*, and *R*- factors based on ALL data will be even larger.

### Fractional atomic coordinates and isotropic or equivalent isotropic displacement parameters (Å^2^)

**Table:**

	*x*	*y*	*z*	*U*_iso_*/*U*_eq_	Occ. (<1)
O1	0.10022 (4)	1.12212 (19)	0.84005 (7)	0.0407 (3)	
O2	0.10797 (4)	0.80068 (19)	0.84277 (7)	0.0389 (3)	
O3	0.17783 (4)	1.0055 (2)	0.75085 (6)	0.0409 (3)	
O4	0.25038 (4)	1.00854 (18)	0.76690 (6)	0.0336 (3)	
N1	0.12908 (5)	0.9423 (2)	0.99876 (7)	0.0313 (3)	
N2	0.28368 (5)	1.0327 (2)	0.90462 (7)	0.0300 (3)	
S2	0.219461 (14)	0.98751 (6)	0.99760 (2)	0.03223 (14)	
C6	0.16602 (6)	0.9698 (2)	0.96274 (9)	0.0306 (4)	
C7	0.16479 (6)	0.9824 (2)	0.89280 (9)	0.0298 (4)	
C8	0.20672 (6)	1.0028 (2)	0.86477 (9)	0.0288 (4)	
C9	0.24011 (6)	1.0096 (2)	0.91492 (8)	0.0290 (4)	
C16	0.12129 (6)	0.9810 (3)	0.85451 (9)	0.0321 (4)	
C17	0.06400 (6)	0.7790 (3)	0.81304 (11)	0.0482 (5)	
H17A	0.0637	0.7996	0.7628	0.058*	
H17B	0.0444	0.8747	0.8330	0.058*	
C18	0.04913 (8)	0.5837 (4)	0.82806 (14)	0.0711 (8)	
H18A	0.0697	0.4902	0.8105	0.107*	
H18B	0.0202	0.5629	0.8060	0.107*	
H18C	0.0476	0.5677	0.8778	0.107*	
C19	0.20984 (6)	1.0069 (2)	0.78909 (9)	0.0302 (4)	
C20	0.25276 (6)	1.0069 (3)	0.69247 (8)	0.0351 (4)	
H20A	0.2385	1.1227	0.6724	0.042*	
H20B	0.2379	0.8918	0.6729	0.042*	
C21	0.30039 (6)	1.0039 (3)	0.67686 (10)	0.0399 (4)	
H21A	0.3148	1.1182	0.6965	0.060*	
H21B	0.3032	1.0032	0.6271	0.060*	
H21C	0.3141	0.8884	0.6968	0.060*	
S1	0.04126 (2)	0.87795 (11)	1.05416 (4)	0.0370 (3)	0.824 (3)
C1	0.01520 (10)	0.8382 (8)	1.1284 (2)	0.0404 (7)	0.824 (3)
H1	−0.0153	0.8193	1.1302	0.049*	0.824 (3)
C2	0.04307 (17)	0.8370 (10)	1.1840 (3)	0.0417 (10)	0.824 (3)
H2	0.0342	0.8170	1.2294	0.050*	0.824 (3)
C3	0.08692 (15)	0.8685 (7)	1.1675 (2)	0.0352 (8)	0.824 (3)
H3	0.1106	0.8741	1.2005	0.042*	0.824 (3)
C4	0.09117 (17)	0.8899 (18)	1.0984 (3)	0.0307 (6)	0.824 (3)
C5	0.1310 (4)	0.925 (5)	1.0640 (5)	0.0317 (7)	0.824 (3)
H5	0.1581	0.9349	1.0894	0.038*	0.824 (3)
C10	0.3117 (3)	1.0332 (16)	0.9566 (10)	0.0321 (10)	0.824 (3)
H10	0.3007	1.0101	1.0005	0.038*	0.824 (3)
C11	0.3573 (2)	1.065 (3)	0.9540 (3)	0.0333 (6)	0.824 (3)
C12	0.38669 (14)	1.0773 (7)	1.01056 (16)	0.0390 (7)	0.824 (3)
H12	0.3798	1.0662	1.0573	0.047*	0.824 (3)
C13	0.42795 (12)	1.1086 (7)	0.98639 (14)	0.0409 (7)	0.824 (3)
H13	0.4545	1.1207	1.0130	0.049*	0.824 (3)
C14	0.42238 (10)	1.1186 (6)	0.91597 (15)	0.0400 (7)	0.824 (3)
H14	0.4451	1.1407	0.8855	0.048*	0.824 (3)
N3	0.38014 (10)	1.0927 (4)	0.89645 (15)	0.0357 (6)	0.824 (3)
C15	0.36240 (9)	1.0926 (4)	0.82465 (15)	0.0449 (6)	0.824 (3)
H15A	0.3479	1.2155	0.8146	0.067*	0.824 (3)
H15B	0.3414	0.9877	0.8182	0.067*	0.824 (3)
H15C	0.3862	1.0747	0.7936	0.067*	0.824 (3)
S1'	0.37454 (15)	1.1247 (7)	0.8684 (3)	0.0464 (14)	0.176 (3)
C1'	0.4267 (4)	1.143 (3)	0.8980 (8)	0.0400 (9)	0.176 (3)
H1'	0.4507	1.1708	0.8707	0.048*	0.176 (3)
C2'	0.4294 (6)	1.113 (4)	0.9662 (8)	0.0407 (9)	0.176 (3)
H2'	0.4559	1.1263	0.9927	0.049*	0.176 (3)
C3'	0.3897 (7)	1.060 (4)	0.9958 (8)	0.0390 (9)	0.176 (3)
H3'	0.3874	1.0219	1.0421	0.047*	0.176 (3)
C4'	0.3551 (9)	1.072 (16)	0.9488 (14)	0.0333 (8)	0.176 (3)
C5'	0.128 (2)	0.92 (2)	1.064 (2)	0.0318 (9)	0.176 (3)
H5'	0.1560	0.9212	1.0873	0.038*	0.176 (3)
C10'	0.3104 (14)	1.053 (8)	0.957 (5)	0.0321 (11)	0.176 (3)
H10'	0.2995	1.0542	1.0014	0.039*	0.176 (3)
C11'	0.0926 (8)	0.881 (9)	1.1072 (13)	0.0307 (8)	0.176 (3)
C12'	0.0912 (8)	0.835 (4)	1.1755 (12)	0.0351 (9)	0.176 (3)
H12'	0.1155	0.8234	1.2073	0.042*	0.176 (3)
C13'	0.0477 (9)	0.809 (5)	1.1897 (12)	0.0416 (11)	0.176 (3)
H13'	0.0362	0.7884	1.2334	0.050*	0.176 (3)
C14'	0.0245 (6)	0.819 (4)	1.1278 (10)	0.0406 (9)	0.176 (3)
H14'	−0.0061	0.8035	1.1212	0.049*	0.176 (3)
N3'	0.0527 (4)	0.8565 (19)	1.0768 (6)	0.041 (3)	0.176 (3)
C15'	0.0380 (4)	0.8571 (17)	1.0042 (6)	0.040 (3)	0.176 (3)
H15D	0.0084	0.8052	0.9994	0.059*	0.176 (3)
H15E	0.0576	0.7776	0.9780	0.059*	0.176 (3)
H15F	0.0382	0.9890	0.9867	0.059*	0.176 (3)

### Atomic displacement parameters (Å^2^)

**Table:**

	*U*^11^	*U*^22^	*U*^33^	*U*^12^	*U*^13^	*U*^23^
O1	0.0392 (7)	0.0416 (8)	0.0411 (7)	0.0086 (6)	0.0006 (6)	0.0000 (6)
O2	0.0287 (7)	0.0390 (7)	0.0488 (8)	−0.0035 (5)	−0.0020 (6)	−0.0017 (6)
O3	0.0338 (7)	0.0578 (9)	0.0313 (7)	−0.0018 (6)	0.0021 (6)	−0.0045 (6)
O4	0.0316 (7)	0.0439 (7)	0.0256 (6)	−0.0015 (5)	0.0058 (5)	−0.0011 (5)
N1	0.0290 (8)	0.0329 (8)	0.0327 (8)	−0.0002 (6)	0.0090 (6)	−0.0012 (6)
N2	0.0288 (8)	0.0312 (8)	0.0302 (7)	−0.0012 (6)	0.0042 (6)	−0.0008 (6)
S2	0.0290 (2)	0.0407 (3)	0.0274 (2)	0.00037 (17)	0.00565 (17)	−0.00015 (17)
C6	0.0293 (9)	0.0304 (9)	0.0325 (9)	−0.0006 (7)	0.0052 (7)	−0.0023 (7)
C7	0.0303 (9)	0.0280 (9)	0.0316 (9)	0.0006 (7)	0.0049 (7)	−0.0006 (7)
C8	0.0294 (9)	0.0282 (9)	0.0292 (8)	0.0003 (6)	0.0057 (7)	−0.0026 (7)
C9	0.0301 (9)	0.0293 (9)	0.0280 (8)	0.0010 (6)	0.0061 (7)	−0.0011 (7)
C16	0.0298 (9)	0.0381 (10)	0.0289 (8)	−0.0020 (7)	0.0080 (7)	−0.0026 (7)
C17	0.0331 (10)	0.0482 (12)	0.0622 (13)	−0.0047 (9)	−0.0093 (9)	0.0014 (10)
C18	0.0580 (16)	0.0732 (18)	0.0798 (18)	−0.0287 (13)	−0.0214 (14)	0.0224 (14)
C19	0.0299 (9)	0.0297 (9)	0.0315 (9)	−0.0018 (7)	0.0046 (7)	−0.0013 (7)
C20	0.0407 (11)	0.0407 (10)	0.0243 (9)	−0.0030 (8)	0.0068 (7)	−0.0017 (7)
C21	0.0411 (11)	0.0450 (11)	0.0346 (10)	−0.0044 (8)	0.0112 (8)	−0.0025 (8)
S1	0.0284 (4)	0.0440 (4)	0.0388 (6)	−0.0025 (3)	0.0032 (3)	−0.0030 (3)
C1	0.0288 (16)	0.0424 (16)	0.0513 (11)	−0.0017 (15)	0.0147 (12)	−0.0097 (10)
C2	0.0397 (15)	0.046 (2)	0.0412 (13)	−0.0025 (14)	0.0167 (11)	−0.0029 (12)
C3	0.0323 (14)	0.039 (2)	0.0350 (15)	0.0005 (12)	0.0062 (10)	0.0008 (12)
C4	0.0291 (10)	0.0334 (15)	0.0299 (16)	−0.0004 (9)	0.0041 (10)	−0.0020 (18)
C5	0.027 (2)	0.0327 (10)	0.0354 (9)	0.000 (2)	0.0052 (10)	−0.0023 (7)
C10	0.0342 (11)	0.031 (3)	0.0311 (9)	0.0010 (13)	0.0056 (9)	0.001 (2)
C11	0.0328 (11)	0.0312 (11)	0.0360 (14)	0.0008 (12)	0.0025 (9)	0.000 (2)
C12	0.0371 (12)	0.0433 (15)	0.0364 (16)	0.0017 (10)	0.0007 (12)	−0.0064 (14)
C13	0.0331 (11)	0.0445 (12)	0.0444 (17)	−0.0007 (9)	−0.0045 (14)	−0.0032 (17)
C14	0.0296 (11)	0.0443 (15)	0.0469 (19)	−0.0015 (9)	0.0115 (11)	0.0047 (13)
N3	0.0340 (12)	0.0396 (14)	0.0336 (14)	0.0007 (9)	0.0031 (12)	0.0051 (11)
C15	0.0393 (14)	0.0672 (18)	0.0285 (14)	0.0014 (12)	0.0060 (11)	0.0073 (12)
S1'	0.036 (2)	0.054 (2)	0.051 (3)	−0.0032 (14)	0.011 (2)	0.002 (2)
C1'	0.0299 (14)	0.0443 (17)	0.047 (2)	−0.0016 (13)	0.0112 (14)	0.0045 (16)
C2'	0.0328 (13)	0.0445 (14)	0.044 (2)	−0.0007 (12)	−0.0038 (17)	−0.003 (2)
C3'	0.0372 (15)	0.0431 (17)	0.0367 (18)	0.0018 (13)	0.0010 (15)	−0.0061 (17)
C4'	0.0328 (13)	0.0312 (13)	0.0358 (16)	0.0009 (15)	0.0020 (12)	−0.001 (2)
C5'	0.028 (2)	0.0327 (13)	0.0353 (12)	0.000 (2)	0.0048 (13)	−0.0023 (11)
C10'	0.0341 (13)	0.031 (3)	0.0312 (12)	0.0011 (15)	0.0055 (12)	0.000 (2)
C11'	0.0288 (12)	0.0333 (17)	0.0303 (18)	−0.0003 (12)	0.0040 (13)	−0.002 (2)
C12'	0.0324 (16)	0.039 (2)	0.0349 (17)	0.0005 (15)	0.0064 (13)	0.0009 (15)
C13'	0.0397 (17)	0.045 (3)	0.0411 (15)	−0.0021 (16)	0.0160 (14)	−0.0028 (14)
C14'	0.0293 (19)	0.0423 (18)	0.0512 (14)	−0.0018 (17)	0.0142 (15)	−0.0094 (13)
N3'	0.041 (4)	0.041 (4)	0.040 (4)	0.001 (3)	−0.003 (3)	0.007 (3)
C15'	0.040 (5)	0.047 (6)	0.032 (6)	−0.005 (4)	−0.006 (4)	0.001 (4)

### Geometric parameters (Å, °)

**Table:**

O1—C16	1.203 (2)	C5—H5	0.9500
O2—C16	1.337 (2)	C10—C11	1.423 (9)
O2—C17	1.453 (2)	C10—H10	0.9500
O3—C19	1.206 (2)	C11—N3	1.366 (5)
O4—C19	1.339 (2)	C11—C12	1.395 (5)
O4—C20	1.4578 (19)	C12—C13	1.391 (4)
N1—C5	1.278 (9)	C12—H12	0.9500
N1—C5'	1.28 (4)	C13—C14	1.379 (4)
N1—C6	1.377 (2)	C13—H13	0.9500
N2—C10'	1.28 (8)	C14—N3	1.347 (4)
N2—C10	1.299 (17)	C14—H14	0.9500
N2—C9	1.373 (2)	N3—C15	1.479 (3)
S2—C6	1.7519 (18)	C15—H15A	0.9800
S2—C9	1.7685 (16)	C15—H15B	0.9800
C6—C7	1.367 (2)	C15—H15C	0.9800
C7—C8	1.431 (2)	S1'—C1'	1.683 (14)
C7—C16	1.500 (2)	S1'—C4'	1.744 (16)
C8—C9	1.385 (2)	C1'—C2'	1.345 (14)
C8—C19	1.485 (2)	C1'—H1'	0.9500
C17—C18	1.468 (3)	C2'—C3'	1.422 (15)
C17—H17A	0.9900	C2'—H2'	0.9500
C17—H17B	0.9900	C3'—C4'	1.374 (17)
C18—H18A	0.9800	C3'—H3'	0.9500
C18—H18B	0.9800	C4'—C10'	1.40 (4)
C18—H18C	0.9800	C5'—C11'	1.44 (4)
C20—C21	1.509 (3)	C5'—H5'	0.9500
C20—H20A	0.9900	C10'—H10'	0.9500
C20—H20B	0.9900	C11'—N3'	1.348 (17)
C21—H21A	0.9800	C11'—C12'	1.374 (18)
C21—H21B	0.9800	C12'—C13'	1.391 (18)
C21—H21C	0.9800	C12'—H12'	0.9500
S1—C1	1.711 (4)	C13'—C14'	1.375 (18)
S1—C4	1.726 (4)	C13'—H13'	0.9500
C1—C2	1.350 (5)	C14'—N3'	1.375 (16)
C1—H1	0.9500	C14'—H14'	0.9500
C2—C3	1.418 (4)	N3'—C15'	1.467 (12)
C2—H2	0.9500	C15'—H15D	0.9800
C3—C4	1.368 (5)	C15'—H15E	0.9800
C3—H3	0.9500	C15'—H15F	0.9800
C4—C5	1.446 (9)		
			
C16—O2—C17	115.94 (15)	N1—C5—C4	118.5 (10)
C19—O4—C20	114.46 (14)	N1—C5—H5	120.7
C5—N1—C6	121.4 (5)	C4—C5—H5	120.7
C5'—N1—C6	126 (3)	N2—C10—C11	126.2 (13)
C10'—N2—C9	119 (3)	N2—C10—H10	116.9
C10—N2—C9	120.1 (6)	C11—C10—H10	116.9
C6—S2—C9	91.28 (8)	N3—C11—C12	107.7 (4)
C7—C6—N1	122.32 (17)	N3—C11—C10	126.6 (9)
C7—C6—S2	111.36 (13)	C12—C11—C10	125.7 (9)
N1—C6—S2	126.31 (13)	C13—C12—C11	107.9 (3)
C6—C7—C8	113.93 (16)	C13—C12—H12	126.0
C6—C7—C16	118.61 (16)	C11—C12—H12	126.1
C8—C7—C16	127.42 (15)	C14—C13—C12	105.9 (3)
C9—C8—C7	112.57 (15)	C14—C13—H13	127.0
C9—C8—C19	128.43 (16)	C12—C13—H13	127.1
C7—C8—C19	118.96 (16)	N3—C14—C13	110.3 (3)
N2—C9—C8	126.55 (15)	N3—C14—H14	124.8
N2—C9—S2	122.60 (13)	C13—C14—H14	124.8
C8—C9—S2	110.85 (13)	C14—N3—C11	108.2 (3)
O1—C16—O2	124.72 (17)	C14—N3—C15	125.1 (3)
O1—C16—C7	124.68 (17)	C11—N3—C15	126.8 (3)
O2—C16—C7	110.46 (15)	C1'—S1'—C4'	94.0 (9)
O2—C17—C18	108.00 (17)	C2'—C1'—S1'	109.9 (11)
O2—C17—H17A	110.1	C2'—C1'—H1'	125.0
C18—C17—H17A	110.1	S1'—C1'—H1'	125.1
O2—C17—H17B	110.1	C1'—C2'—C3'	115.3 (14)
C18—C17—H17B	110.1	C1'—C2'—H2'	122.3
H17A—C17—H17B	108.4	C3'—C2'—H2'	122.3
C17—C18—H18A	109.5	C4'—C3'—C2'	111.5 (15)
C17—C18—H18B	109.5	C4'—C3'—H3'	124.3
H18A—C18—H18B	109.5	C2'—C3'—H3'	124.2
C17—C18—H18C	109.5	C3'—C4'—C10'	131 (4)
H18A—C18—H18C	109.5	C3'—C4'—S1'	108.9 (14)
H18B—C18—H18C	109.5	C10'—C4'—S1'	120 (4)
O3—C19—O4	122.97 (16)	N1—C5'—C11'	131 (5)
O3—C19—C8	121.73 (16)	N1—C5'—H5'	114.2
O4—C19—C8	115.29 (15)	C11'—C5'—H5'	114.3
O4—C20—C21	107.25 (15)	N2—C10'—C4'	121 (7)
O4—C20—H20A	110.3	N2—C10'—H10'	119.7
C21—C20—H20A	110.3	C4'—C10'—H10'	119.9
O4—C20—H20B	110.3	N3'—C11'—C12'	109.1 (17)
C21—C20—H20B	110.3	N3'—C11'—C5'	118 (3)
H20A—C20—H20B	108.5	C12'—C11'—C5'	132 (3)
C20—C21—H21A	109.5	C11'—C12'—C13'	107.3 (16)
C20—C21—H21B	109.5	C11'—C12'—H12'	126.4
H21A—C21—H21B	109.5	C13'—C12'—H12'	126.3
C20—C21—H21C	109.5	C14'—C13'—C12'	106.5 (16)
H21A—C21—H21C	109.5	C14'—C13'—H13'	126.8
H21B—C21—H21C	109.5	C12'—C13'—H13'	126.8
C1—S1—C4	91.4 (2)	N3'—C14'—C13'	109.0 (15)
C2—C1—S1	112.1 (3)	N3'—C14'—H14'	125.5
C2—C1—H1	123.9	C13'—C14'—H14'	125.5
S1—C1—H1	123.9	C11'—N3'—C14'	107.4 (14)
C1—C2—C3	113.0 (4)	C11'—N3'—C15'	131.1 (14)
C1—C2—H2	123.5	C14'—N3'—C15'	121.5 (13)
C3—C2—H2	123.5	N3'—C15'—H15D	109.5
C4—C3—C2	112.2 (4)	N3'—C15'—H15E	109.5
C4—C3—H3	123.9	H15D—C15'—H15E	109.5
C2—C3—H3	123.9	N3'—C15'—H15F	109.5
C3—C4—C5	126.8 (6)	H15D—C15'—H15F	109.5
C3—C4—S1	111.3 (3)	H15E—C15'—H15F	109.5
C5—C4—S1	121.9 (6)		
			
C5—N1—C6—C7	177.8 (17)	C1—S1—C4—C3	−1.4 (8)
C5'—N1—C6—C7	175 (9)	C1—S1—C4—C5	−179.5 (18)
C5—N1—C6—S2	−0.6 (17)	C5'—N1—C5—C4	−27 (91)
C5'—N1—C6—S2	−3(9)	C6—N1—C5—C4	−177.9 (15)
C9—S2—C6—C7	−0.63 (13)	C3—C4—C5—N1	−179.7 (16)
C9—S2—C6—N1	177.95 (16)	S1—C4—C5—N1	−2(3)
N1—C6—C7—C8	−177.47 (15)	C9—N2—C10—C11	−176.7 (13)
S2—C6—C7—C8	1.18 (19)	N2—C10—C11—N3	−3(3)
N1—C6—C7—C16	4.7 (2)	N2—C10—C11—C12	175.7 (13)
S2—C6—C7—C16	−176.64 (12)	N3—C11—C12—C13	−1.3 (17)
C6—C7—C8—C9	−1.3 (2)	C10—C11—C12—C13	179.8 (16)
C16—C7—C8—C9	176.33 (16)	C11—C12—C13—C14	1.2 (11)
C6—C7—C8—C19	176.62 (15)	C12—C13—C14—N3	−0.7 (5)
C16—C7—C8—C19	−5.8 (3)	C13—C14—N3—C11	−0.1 (11)
C10'—N2—C9—C8	174 (3)	C13—C14—N3—C15	−179.4 (3)
C10—N2—C9—C8	−178.8 (6)	C12—C11—N3—C14	0.8 (16)
C10'—N2—C9—S2	−5(3)	C10—C11—N3—C14	179.7 (17)
C10—N2—C9—S2	2.1 (6)	C12—C11—N3—C15	−179.8 (7)
C7—C8—C9—N2	−178.42 (16)	C10—C11—N3—C15	−1(3)
C19—C8—C9—N2	4.0 (3)	C4'—S1'—C1'—C2'	−1(4)
C7—C8—C9—S2	0.73 (19)	S1'—C1'—C2'—C3'	4(3)
C19—C8—C9—S2	−176.89 (14)	C1'—C2'—C3'—C4'	−7(6)
C6—S2—C9—N2	179.12 (15)	C2'—C3'—C4'—C10'	−173 (9)
C6—S2—C9—C8	−0.07 (13)	C2'—C3'—C4'—S1'	6(8)
C17—O2—C16—O1	−2.7 (3)	C1'—S1'—C4'—C3'	−3(7)
C17—O2—C16—C7	173.15 (15)	C1'—S1'—C4'—C10'	176 (8)
C6—C7—C16—O1	90.3 (2)	C5—N1—C5'—C11'	151 (100)
C8—C7—C16—O1	−87.2 (2)	C6—N1—C5'—C11'	−178 (10)
C6—C7—C16—O2	−85.62 (19)	C9—N2—C10'—C4'	178 (6)
C8—C7—C16—O2	96.9 (2)	C3'—C4'—C10'—N2	−167 (8)
C16—O2—C17—C18	−158.52 (19)	S1'—C4'—C10'—N2	15 (11)
C20—O4—C19—O3	−0.5 (2)	N1—C5'—C11'—N3'	5(19)
C20—O4—C19—C8	178.27 (14)	N1—C5'—C11'—C12'	173 (10)
C9—C8—C19—O3	−177.86 (18)	N3'—C11'—C12'—C13'	−9(5)
C7—C8—C19—O3	4.7 (2)	C5'—C11'—C12'—C13'	−177 (10)
C9—C8—C19—O4	3.4 (2)	C11'—C12'—C13'—C14'	6(4)
C7—C8—C19—O4	−174.11 (15)	C12'—C13'—C14'—N3'	−2(4)
C19—O4—C20—C21	−178.59 (14)	C12'—C11'—N3'—C14'	8(5)
C4—S1—C1—C2	0.8 (6)	C5'—C11'—N3'—C14'	178 (8)
S1—C1—C2—C3	0.0 (7)	C12'—C11'—N3'—C15'	−170 (2)
C1—C2—C3—C4	−1.0 (9)	C5'—C11'—N3'—C15'	1(10)
C2—C3—C4—C5	179.6 (19)	C13'—C14'—N3'—C11'	−4(4)
C2—C3—C4—S1	1.6 (10)	C13'—C14'—N3'—C15'	174 (2)
